# Nonlinear amplification of microwave signals in spin-torque oscillators

**DOI:** 10.1038/s41467-023-37916-9

**Published:** 2023-04-17

**Authors:** Keqiang Zhu, Mario Carpentieri, Like Zhang, Bin Fang, Jialin Cai, Roman Verba, Anna Giordano, Vito Puliafito, Baoshun Zhang, Giovanni Finocchio, Zhongming Zeng

**Affiliations:** 1 Nanofabrication facility, Suzhou Institute of Nano-Tech and Nano-Bionics, Chinese Academy of Sciences, Suzhou, Jiangsu China; 2 Department of Electrical and Information Engineering, Politecnico di Bari, Bari, Italy; 3 School of Electronics and Information Engineering, Wuxi University, Wuxi, Jiangsu China; 4 Institute of Magnetism, Kyiv, Ukraine; 5 Department of Engineering, University of Messina, Messina, Italy; 6 Department of Mathematical and Computer Sciences, Physical Sciences and Earth Sciences, University of Messina, Messina, Italy; 7 Division of Nano-Devices and Technologies & Nanchang Key Laboratory of Advanced Packaging, Jiangxi Institute of Nanotechnology, Nanchang, China

**Keywords:** Magnetic devices, Electrical and electronic engineering

## Abstract

Spintronics-based microwave devices, such as oscillators and detectors, have been the subject of intensive investigation in recent years owing to the potential reductions in size and power consumption. However, only a few concepts for spintronic amplifiers have been proposed, typically requiring complex device configurations or material stacks. Here, we demonstrate a spintronic amplifier based on two-terminal magnetic tunnel junctions (MTJs) produced with CMOS-compatible material stacks that have already been used for spin-transfer torque memories. We achieve a record gain (|*S*_11_ | > 2) for input power on the order of nW (<−40 dBm) at an appropriate choice of the bias field direction and amplitude. Based on micromagnetic simulations and experiments, we describe the fundamental aspects driving the amplification and show the key role of the co-existence in microwave emissions of a dynamic state of the MTJ excited by a dc current and the injection locking mode driven by the microwave input signal. Our work provides a way to develop a class of compact amplifiers that can impact the design of the next generation of spintronics-CMOS hybrid systems.

## Abstract (web summary)

Microwave devices are instrumental in wireless communications. Recently, spintronic-based microwave devices have seen significant interest, with the potential for smaller size, and lower power consumption. Here, Zhu et al demonstrate a spintronic amplifier with record gain, which uses material stacks already employed in industrially fabricated magnetic memories.

## Introduction

The discovery of the giant-magnetoresistive effect and spin-transfer torque enabled the birth of spintronics, allowing devices to take advantage of the spin together with charge. Such a technology has the potential to impact the design of radio-frequency and microwave devices, improving their performance in terms of power consumption and compactness^1^. Amplifiers serve as key elements in radio frequency circuits, however, with spintronic technology still in its infancy, no clear strategy for the development of spintronic amplifiers has emerged.

The concept of microwave amplification based on spin-transfer torque in a three-terminal device was proposed by Slonczewski in one of his patents^2^. Since then, spintronic amplifiers have been realized with feedback mechanisms^3,4^, by combining magnetic tunnel junctions (MTJs) with electrically isolated metallic wires in a scheme known as spin transistor^5^ or exciting MTJ resonance with a microwave field^6^. Recently microwave amplification has been demonstrated with two-terminal MTJs biased with a direct current (dc) and designed with materials that have a large heat-to-spin conversion^7^. For those two-terminal devices, the amplification gain can be expressed by the *S*_11_ parameter^7,8^:

1 $${\bar{S}}_{11}(f)=\frac{{\bar{Z}}_{{{{{{\rm{MTJ}}}}}}}(f)-{\bar{Z}}_{0}}{{\bar{Z}}_{{{{{{\rm{MTJ}}}}}}}(f)+{\bar{Z}}_{0}}$$

where ${\bar{Z}}_{{{{{{\rm{MTJ}}}}}}}$ is the complex value of the MTJ impedance for a given frequency *f* and *Z*_0_ is the 50 Ω characteristic impedance of the connecting microwave line. Spintronic devices based on MTJs have shown potential impact in technological solutions that are CMOS-compatible^9^, such as spin-transfer torque (STT) memories^10^, detectors^11,12^, and oscillators^13–15^. STT oscillators are strongly nonlinear and exhibit rich behavior, including injection locking^16^, fractional^17^ and hysteretic synchronization^18,19^. In particular, injection locking has an important role in developing spin diodes with a very high sensitivity (output dc voltage over input microwave power)^20^, overcoming the thermodynamic limit of semiconductor counterparts^11,12,21^, and exceeding 4 MV/W when combined with the spin-bolometric effect^22^. This high sensitivity arises from the nonlinear detection term due to the partial injection-locking driven by a trade-off between the microwave external source and the magnetic noise^12,23^. Injection locking is already employed in CMOS technologies^24^ and optics^25^ for the development of high-gain amplifiers.

Here, we present a strategy to design nonlinear spintronic amplifiers exhibiting a large amplification (${\bar{S}}_{11}\left(f\right) \, > \, 2$) driven by STT at an incident power of −60 dBm (1 nW). The amplifiers were produced with the standard material stacks used for spin-transfer-torque magnetic random access memory (STT-MRAM) and do not need MTJs with specific materials that have an efficient heat-to-spin conversion^7^. The key ingredients to have amplification are: (i) a dc bias voltage larger than a critical value *V*_c_ (the MTJ should work in the active regime as a spin-transfer torque oscillator (STO)), (ii) a bias field applied along a direction that minimizes the current tunability of the oscillation frequency of the STO, and (iii) the partial injection locking with the co-existence of two modes. In particular, we systematically measured the |*S*_11_ | parameter as a function of the field direction and amplitude, finding the best amplification performance for a field *H*_ext_ of 60 mT applied along the directions in the polar angle ${\theta }_{0}={30}^{\circ }$ and azimuthal angle ${\varphi }_{0}={90}^{\circ }$, the direction at which the tunability of the microwave emissions was zero and the oscillation frequency did not depend on the dc voltage. Micromagnetic simulations and experimental measurements showed that the amplification at low input microwave powers arose from the partial synchronization between the input microwave signal and the dynamical state of the STO, i.e., self-oscillation, through the mechanism of injection locking. In addition, time-resolved voltage traces have been measured to study the phase stability of the oscillator.

## Results

### Microwave amplification with | *S*_11_ | measurement

**Fig. 1:**
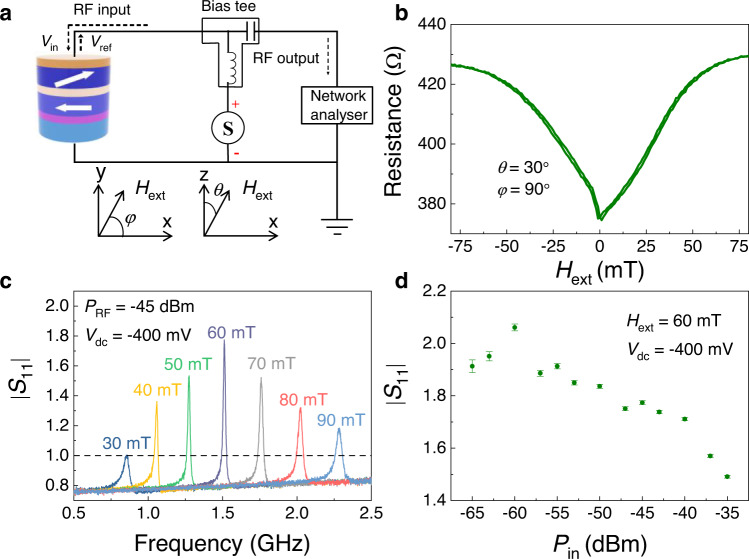
Microwave amplification with | *S*_11_ | measurement. **a** A schematic diagram of the measurement systems. Inset: Cartesian coordinate system, with a description of the direction of the applied magnetic field (*H*_ext_) and the indication of polar *θ* and azimuthal angle *φ*. *V*_in_ and *V*_ref_ are the input and output voltage, respectively. The long and short in-plane axes of the MTJ coincide with the *x* and *y*-axes, respectively. For the measurements, the field is applied along the *θ* = 30° and *φ* = 90° directions. **b** Magnetoresistance-field curves of one MTJ device for *I*_dc_ = 10 μA at azimuthal angle *θ* = 30° and polar angle *φ* = 90°. **c** | *S*_11_ | as a function of frequency for a bias voltage *V*_dc_ = −400 mV and RF power *P*_RF_ = −45 dBm under different values of external field ranging from 30 to 90 mT. **d** | *S*_11_ | as a function of the input microwave power *P*_in_ (*H*_ext_ = 60 mT and *V*_dc_ = −400 mV). To avoid the influence of noise, the error bars were added by estimating the average and standard deviation of the data.

The multilayer samples were deposited using magnetron sputtering onto oxidized Si wafers and then annealed at 300 °C for 2.0 h under an in-plane magnetic field of 1 T. The MTJ samples had a layered structure (the numbers in parentheses are in nm): a bottom contact, an exchange-biased synthetic antiferromagnetic polarizer layer comprising PtMn(15)/Co_70_Fe_30_ (2.3)/Ru (0.85)/Co_40_Fe_40_B_20_ (2.4), a tunnel barrier comprising MgO (0.8), a free layer comprising Co_20_Fe_60_B_20_ (1.65), and a top contact. The thin films were patterned into elliptical MTJ nanopillars (130 nm × 60 nm). A sketch of the device is shown in Fig. 1a, where the magnetization configurations of the polarizer and free layer together with a schematic of the measurement system are also included (see Methods). The ac and dc voltages were applied to the device through a bias tee. We characterized the output microwave voltage in the MTJ through |*S*_11_ | parameter measurements using a vector network analyzer. All data in this work were measured at room temperature. The reference Cartesian coordinate system, with the indication of polar angle *θ* and azimuthal angle *φ*, is shown as the inset of Fig. 1a, where the *x*-axis is defined to be parallel to the long axis of the elliptical nanopillar and the *z*-axis is the out-of-plane direction. The iron-rich Co_20_Fe_60_B_20_ free layer exhibited interfacial perpendicular magnetic anisotropy (PMA)^26^ which was designed to partially compensate for the out-of-plane demagnetizing field^12^. The hysteresis loops from the magnetoresistance as a function of the in-plane and perpendicular magnetic fields are shown in Supplementary Fig. 1. The experimental saturation magnetization *M*_S_ was 9.5 × 10^5^ Am^−1^, and the resistances in the parallel *R*_P_ and antiparallel *R*_AP_ states were 355 and 668 Ω, respectively. The tunneling magnetoresistive ratio, defined as (*R*_AP_-*R*_P_)/*R*_P,_ was larger than 85%. Figure 1b shows the field scan of the magnetoresistive signal as a function of the field amplitude applied along the ${\theta }_{0}={30}^{\circ }$ and ${\varphi }_{0}={90}^{\circ }$ directions, where the maximum amplification performance for this device is achieved. For |*H*_ext_ | above 50 mT, the magnetization is almost aligned with the field, and the resistance tended to saturate at 428 Ω.

Figure 1c summarizes |*S*_11_ | as a function of the input frequency for a microwave power *P*_in_ of −45 dBm at a bias voltage *V*_dc_ of −400 mV. The amplification region (|*S*_11_ | > 1) is resonant in nature and is observed for a field amplitude larger than 30 mT and at an input bias voltage larger than the threshold value (see discussion below). The maximum value of |*S*_11_ | = 1.8 is observed at 1.5 GHz for the bias field of 60 mT. The peak position and the frequency range where |*S*_11_ | > 1 is tunable with the external field (25 MHz/mT), as one would expect for the behavior of a resonance mode of saturated ferromagnetic sample. The presence of amplification regions is robust and it has been observed in several devices as shown for example in Supplementary Figs. 2 and 3.

Figure 1d shows |*S*_11_ | as a function of the input power for *V*_dc_ = −400 mV and *H*_ext_ = 60 mT (the field value showing the maximum |*S*_11_ | , see Fig. 1c). The main result here is the observation of a nonlinear dependence of the amplification rate. A variation of the input power does not induce a proportional change of the output power; hence, |*S*_11_ | is not constant but is a nonmonotonic function of the input power, which exhibits a record |*S*_11_ | larger than 2 for *P*_in_ = −60 dBm. In other words, being the amplification power dependent, we point out that this is a nonlinear amplifier which can have a key role in hybrid CMOS-spintronic systems. Supplementary Fig. 4 shows a comparison of |*S*_11_ | as a function of frequency (*V*_dc_ = −400 mV, *H*_ext_ = 600 Oe) for three values of external microwave, −60, −50, and −35 dBm.

**Fig. 2:**
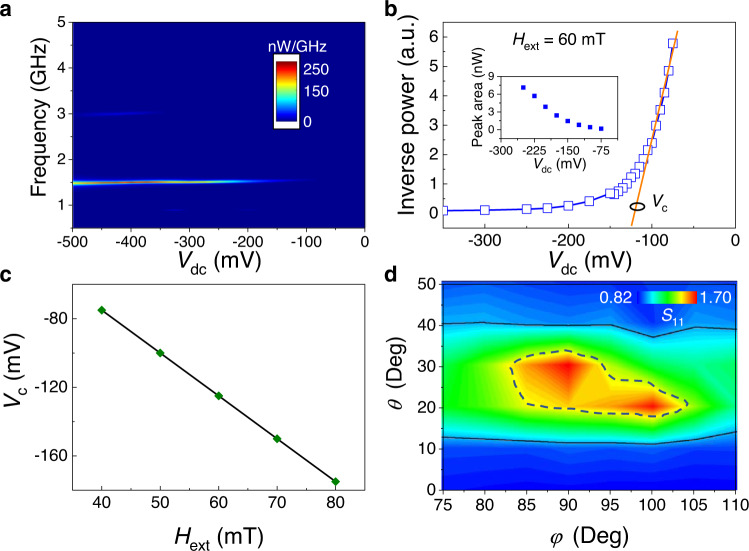
Microwave emissions and amplification dependence of the MTJ. **a** Microwave emission driven by the bias voltage *V*_dc_. **b** Inverse microwave power as a function of the input voltage and the linear approximation at small input power (orange curve). The critical voltage *V*_c_ can be computed from the intersection of the orange curve with the voltage axis, which gives a value of −125 mV for this field configuration. Inset. Microwave power computed as the integral of the excited mode (peak area) as a function of *V*_dc_. **c** Critical voltage as a function of the field amplitude. **d** Phase diagram of |*S*_11_ | as a function of azimuthal *θ* and polar angles *φ* for a fixed field amplitude *H*_ext_ = 60 mT (*V*_dc_ = −450 mV and *P*_in_ = −45 dBm). The solid black line shows the region where the amplification |*S*_11_ | > 1 takes place while the region where |*S*_11_ | > 1.5.

To shed light on the nature of the observed amplification effect, we performed a series of experiments applying only a dc bias to the MTJ (i.e., no incident microwave signal) and measuring the spectra of the output signal. A summary of these experiments, shown in Fig. 2a, demonstrates microwave emissions of the MTJ at negative voltages below a certain threshold. In other words, MTJ behaves as an STO in the active regime. From the voltage dependence of the inverse microwave power^27^, computed as the integral of the excited mode (peak area), we find the threshold voltage for microwave emission to be −125 mV (Fig. 2b). At large negative voltages, the emission spectra show another peak at approximately 3 GHz (see Fig. 2a), which is the second harmonics appearing due to a complex trajectory of the magnetization motion. Figure 2c shows the critical voltage as a function of the external field amplitude ranging from 40 to 80 mT, which demonstrates a natural increase in magnitude due to an increase in the eigenfrequency of the resonance mode and, consequently, of the Gilbert losses.

We wish to stress that for all the configurations studied here, the amplification occurs above the threshold voltages of the microwave emissions. Thus, the amplification takes place when the MTJ works in the active regime as an STO^28^, and the underlying mechanism of the amplification is the partial injection locking of the MTJ self-oscillations to an external microwave signal.

The microwave emissions spectra have been computed by subtracting the microwave emissions measured in presence of the microwave input to the one measured when both bias voltage and microwave input are applied simultaneously (see Supplementary Fig. 5a, b). It can be clearly observed that at low input power there are two peaks (first harmonics of the self-oscillation itself and the mode at the frequency of the input microwave current). On the other hand, at power larger than −37 dBm, it can be observed a significant enhancement in the second harmonics data (see Supplementary Fig. 5b) that is a clear indication of the partial injection locking regime for the reflected microwave voltage with potentially the presence of a parametric effect^29^. In addition, microwave emissions indicate that the amplified signal is not intermittent, in fact the frequency of the jumps between the two modes when those are nonstationary is characterized by a low-frequency mode (see Fig. 6d of ref. ^30^ which is not observed here.

We wish to stress that while the injection locking is a standard phenomenon which may be considered as a way for amplification of microwave signals, in our devices, the injection locking is not the only important phenomenon, moreover, the maximum amplification rate is achieved in the range of input powers, where full injection locking is not achieved. The theoretical basis of the amplification based on this mechanism can be found ahead in the text.

In the experiments with microwave input, we also find that the amplification mechanism is sensitive to the variation of the field angle. Figure 2d summarizes the |*S*_11_ | for *H*_ext_ = 60 mT (*V*_dc_ = −450 mV and *P*_in_ = −45 dBm) as a function of the azimuthal and polar angles. The solid black line in the figure indicates the region of the angles where the amplification is observed |*S*_11_ | > 1 while the dot line indicates the region with |*S*_11_ | > 1.5. The last region spans 20° < *θ* < 30° and 80° < *φ* < 105°. The asymmetry with respect to *φ* = 90°, which may be unexpected, is a consequence of a weak stray dipolar field from the pinned layer. The dependence on the polar angle *φ* is related to the angular dependence of the dynamic magnetoresistance. Oscillations of the resistance at the frequency of the magnetization oscillations (the first harmonic) are proportional to the *m*_*x*_ dynamic component (since magnetization of the pinned layer is along the *x*-axis), and this component is maximized if *φ* = 90°. In contrast, at *φ* = 0°, *m*_*x*_ oscillates at the double frequency (second harmonic) and cannot contribute to the amplification.

The nature of the sensitivity to the azimuthal angle *θ* is more involved. There are several characteristics of STO, which depend on *θ* and, thus, could contribute to the angular dependence of the amplification rate. One of these factors is the effective driving force produced by the microwave current via the spin-transfer torque effect. In our geometry, this force is proportional to the dynamical magnetization component *m*_ξ_, which is perpendicular to the *x*-axis and static magnetization. Although *m*_ξ_ clearly demonstrates a certain dependence on the angle *θ* (i.e., precession ellipticity of the linear eigenmode of the free layer is varied), this dependence is quite smooth and does not have any peculiarities in the 20° < *θ* < 30° range. The contribution from the voltage-controlled magnetic anisotropy, which could exist in such structures, is proportional to *sin*[2*θ*] and cannot explain the observed features.

The last characteristic is the nonlinear frequency shift *N*, which is known to significantly affect the dynamics of STO. In particular, a large nonlinear frequency shift allows to achieve the phase-locking band to an external source that is much wider than that for an isochronous (linear in frequency) oscillator, as well as easily realize the mutual phase-locking of spin-torque oscillators despite a substantial spread of eigenfrequencies^31^. In contrast, the nonlinear frequency shift is not desirable for STO working as an amplifier. The power of the STO in the presence of an external driving force *F* is approximately equal to $p/{p}_{0}=1+F/\scriptstyle\sqrt{1+{(N/\varGamma \xi )}^{2}}$, where $\xi=I/{I}_{{{{{{\rm{th}}}}}}}$ is the supercriticality of the dc bias current (or voltage), and ${p}_{0}$ is the emission power in the absence of a microwave input^27^. As maximal values of *N* are 1-2 orders of magnitude larger than the damping rate *Γ*, the ratio *N*/*Γ* could reach high values of approximately 50−100, resulting in a pronounced dependence of the STO response to an external source with a maximum power at the vanishing nonlinear frequency shift *N* = 0^31^. In addition, a high value of *N* leads to the increased phase noise of STO generation and an increased sensitivity of STO to thermal noise^31,32^, which, finally, could decrease the coherence of the output signal with the input signal.

The nonlinear frequency shift is highly dependent on the magnetization angle, and it is negative for in-plane magnetization, positive when perpendicular to the plane (or vice versa if a ferromagnetic layer exhibits a large perpendicular anisotropy with *Q*-factor *Q* > 1), and equal to zero at certain angles *θ* ≠ 0, *π*/2. Spectra of free-running generation (Fig. 2a) demonstrate that the nonlinear frequency shift vanishes in the range where the amplification takes place; the generation frequency is almost independent of the bias voltage^33^. Thus, the reason why the MTJ should be biased by an external field along a tilted out-of-plane direction with the in-plane component along the hard in-plane axis, near the ${\theta }_{0}={30}^{\circ }$ and ${\varphi }_{0}={90}^{\circ }$ directions in this device geometry, is related to obtaining a nonlinear frequency shift that is as small as possible.

### The time domain scheme and measurements

To study the phase stability of the spintronic amplifier, we have used a different measurement setup where a 4-channel oscilloscope is added to characterize the time-resolved voltage traces (See Supplementary Fig. 6, Supplementary Note 1 and methods). First, we have measured the time-domain traces related to the microwave emissions originated by the STO. To do that, we turned off the signal generator and applied a direct current *I*_dc_ = −0.46 mA (*H*_ext_ = 90 mT, *θ* = 30° and *φ* = 90°), this is the condition where the larger |*S*_11_ | for this device has been found. The time-resolved voltage traces caused by the oscillating magnetoresistance are shown in Supplementary Fig. 7a. Then, the oscillator phase is calculated with zero crossing of the time traces as introduced in ref. ^34^. The time evolution of oscillator phase illustrates some typical characteristics of the STO such as random walk character of the phase variations^34^. As expected, the phase noise dominates the spectral line broadening of STO in free-running regime influencing drastically the coherence of the microwave signal^35^.

**Fig. 3:**
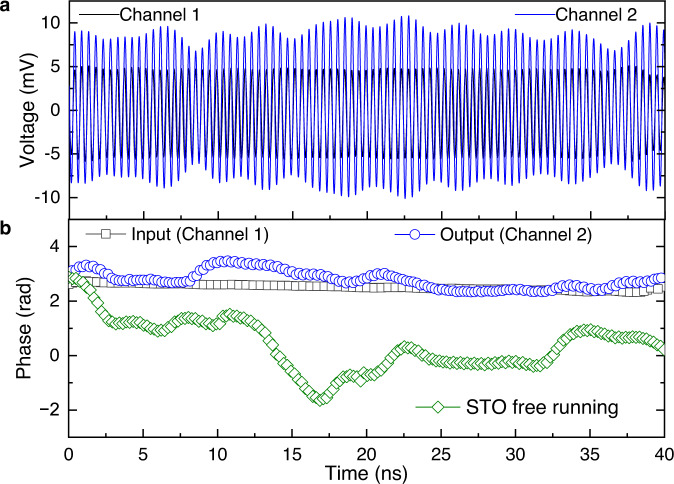
The time domain scheme and measurements. **a** Time-resolved voltage traces of the input signal in channel 1(black line), and the output signal in channel 2 (blue line). Note that we numerically applied a 600 MHz band-pass filter centered at peak frequency (2.1 GHz) to eliminate the background noise, low-frequency noise, and related harmonics. **b** Time-resolved phase traces of input signal (channel 1, black square), output signal (channel 2, blue circle) and STO free running (green square). The phase traces were calculated by the zero-crossing approach as described in Supplementary Note 1.

In the second part of the experiment, we have characterized the time domain traces across the MTJ acting as spintronic amplifier and the input RF signal simultaneously (See Supplementary Fig. 6 and Supplementary Note 1). The RF signal generated by a signal generator was divided into two RF signals (RF_1_ and RF_2_) by the power divider. RF_1_ and RF_2_ have the same frequency, amplitude, and phase. RF_1_ signal is injected into channel 1 of the oscilloscope. RF_2_ signal is the RF input signal applied into the MTJ through a directional coupler and bias tee. The time-resolved voltage (RF output) was measured and displayed in the channel 2 of the oscilloscope through the bias tee and directional coupler, as shown in Supplementary Fig. 6. Figure 3a shows a comparison of the time-resolved voltage traces measured in the channels 1 and 2 of the oscilloscope. The RF_1_ (black line) represents the input signal in channel 1, while channel 2 (blue line) represents the amplified voltage. The two signals have the same central frequency (about 2.1 GHz).

The phase coherence has been evaluated considering zero crossing of the input and output RF signal (see Supplementary Note 1)^34^. Figure 3b shows the phase deviations for a span of 40 ns for the input signal (channel 1, black square) and the output signal (channel 2, blue circle). For a comparison, we have also included the oscillator phase of the STO in the free-running (Fig. 3b, green square). As expected, the phase in the channel 1 remains nearly constant at about 2.6 rad because of the high quality of the input signal. On the other hand, a phase fluctuation can be observed in the time trace measured in the channel 2, while being partially constant from 3 to 11 ns and from 25 ns to 32 ns. However, compared to the oscillator phase of free-running STO, the phase fluctuation of output signal is significantly reduced.

We wish to highlight this phase coherent is achieved with a single device with no feedback in this work. Actually, the coherence in STO has been demonstrated that could be significantly improved by a phase-locked loop circuit^36^, a proven technology in industrial applications. In the ref. ^36^, the linewidth of the free running oscillator (∆*f* = 4.1 MHz) can be reduced to less than 1 Hz under the coupling with a phase-locked loop circuit. This suggests a direction to improve the phase coherence of spintronic amplifiers.

## Discussion

The current applied to the MTJ consists of a dc bias current and ac drive current,

2 $$I={I}_{{{{{{\rm{dc}}}}}}}+{I}_{{{{{{\rm{ac}}}}}}}{{\sin }}\left[{\omega }_{{{{{{\rm{ac}}}}}}}t\right]$$

The resistance of the MTJ is

3 $$R={R}_{0}+\Delta {R}_{{{{{{\rm{dc}}}}}}}\left({I}_{{{{{{\rm{ac}}}}}}}\right)+\Delta {R}_{{{{{{\rm{ac}}}}}}}{{\sin }}\left[{\omega }_{{{{{{\rm{ac}}}}}}}t+\phi \left({\omega }_{{{{{{\rm{ac}}}}}}}\right)\right]$$

In addition to common resistance oscillation at the frequency of magnetization oscillations (the last contribution of Eq. 3), there is a change to the dc resistance, caused by the magnetization oscillations (second term of Eq. 3). Therefore, the total change in ac voltage (at *f*_ac_) due to magnetization oscillations is

4 $$\Delta {V}_{{{{{{\rm{ac}}}}}}}={I}_{{{{{{\rm{dc}}}}}}}\Delta {R}_{{{{{{\rm{ac}}}}}}}{{\sin }}\left[{\omega }_{{{{{{\rm{ac}}}}}}}t+\phi \left({\omega }_{{{{{{\rm{ac}}}}}}}\right)\right]+{I}_{{{{{{\rm{ac}}}}}}}\Delta {R}_{{{{{{\rm{dc}}}}}}}\left({I}_{{{{{{\rm{ac}}}}}}}\right){{\sin }}\left[{\omega }_{{{{{{\rm{ac}}}}}}}t\right]$$

The first term of the equation is proportional to $\Delta {R}_{{{{{{\rm{ac}}}}}}}$ which is related the oscillation power and hence directly related to the injection locking. On the other hand, the second term is proportional to $\Delta {R}_{{{{{{\rm{dc}}}}}}}$ which is originated by the fact that the oscillation axis of the dc dynamics (self-oscillation) can change as a function of ${I}_{{{{{{\rm{ac}}}}}}}$. Here, microwave emissions and micromagnetic simulations show that both contributions are present pointing out that the origin of this high |S_11_ | value is originated by the contribution of both terms. An important role in the amplification is also given by the direction of the polarizer which introduces a phase shift of 180° between the microwave current and voltage having the spin-transfer-torque opposite sign then the charge current, this scenario is the same as the previous experiments published in ref. ^7^ while the amplification mechanism is different, i.e., heat-driven spin torque vs partial injection locking. We additionally would like to highlight that dc (rectified voltage) is given by

5 $$\Delta {V}_{{{{{{\rm{dc}}}}}}}=\frac{1}{2}{I}_{{{{{{\rm{ac}}}}}}}\Delta {R}_{{{{{{\rm{ac}}}}}}}{{\cos }}\left[\phi \left({\omega }_{{{{{{\rm{ac}}}}}}}\right)\right]+{I}_{{{{{{\rm{dc}}}}}}}\Delta {R}_{{{{{{\rm{dc}}}}}}}\left({I}_{{{{{{\rm{ac}}}}}}}\right)$$

As Eq. (5) has, obviously, different structure than Eq. (4), it is clear, that the results on the spin-torque diodes cannot be directly transferred to the case of spin-torque amplifiers.

**Fig. 4:**
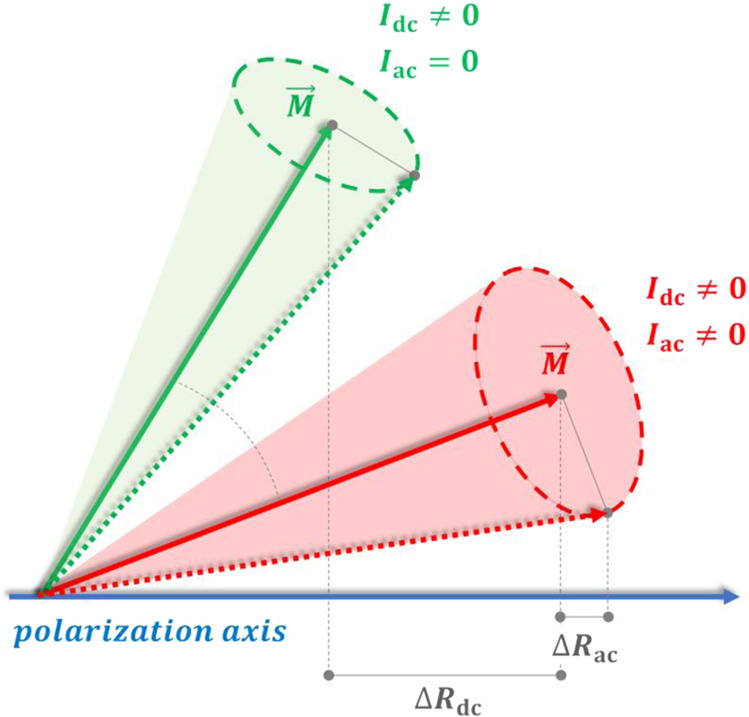
A near-cartoonish depiction of the two mechanisms originating the amplification in the MTJ at low input power. The *I*_dc_ drives the self-oscillation state with an average magnetization ***M*** that changes in presence of the additional *I*_ac_. The difference of the two projections of the average magnetization into the polarization axis give rise to the *ΔR*_dc_$.$ Additionally, the *I*_ac_. changes the amplitude of the oscillating resistance *ΔR*_ac_.

Figure 4 shows a near-cartoonish description of the mechanisms originating the amplification in our MTJs at low input power. Basically, the projection of the oscillation axis in presence and absence of the microwave input is different, thus originating the second contribution of the Eq. 4. On the other hand, there is an oscillation mode at the same frequency of the input microwave signal which gives rise to the contribution related to the first term of Eq. 4.

Thus, our results demonstrate a new operation mode of spin-torque oscillators as amplifiers beyond the phase-locked state. Practical importance of this result is that at small input power and/or large noise (i.e., small devices, room temperature) phase-locked state is often hard to achieve.

To study the amplification mechanism from a theoretical point of view and then estimate ${\bar{Z}}_{{{{{{\rm{MTJ}}}}}}}$ and ${\bar{S}}_{11}\left(f\right)$, we also performed micromagnetic simulations (see Methods for details about the model and the simulation parameters). In the injection locking regime or partial locking, the resistance oscillation is characterized by a component oscillating at the same frequency ${f}_{0}$ of the microwave input. The applied current can be expressed as ${I}_{{{{{{\rm{dc}}}}}}}+{I}_{{{{{{\rm{M}}}}}}}{e}^{-j\left(2\pi {f}_{0}t+{\it\varPhi }_{{{{{{\rm{I}}}}}}}\right)}$, where *I*_dc_ is computed as the ratio between *V*_dc_ and the device resistance at zero microwave input ${R}_{{{\rm{dc}}}}(0)$, where ${I}_{{{{{{\rm{M}}}}}}}$ and $\it {\varPhi }_{{{{{{\rm{I}}}}}}}$ are the microwave current amplitude and its phase, respectively. The resistance is given by *R = R*_dc_(*f*_0_) + *R*_M_${e}^{-j\left(2\pi {f}_{0}t+{\it\varPhi }_{{{{{{\rm{R}}}}}}}\right)}$, where ${R}_{{{{{{\rm{M}}}}}}}$ and $\it {\varPhi }_{{{{{{\rm{R}}}}}}}$ are the amplitude and the phase of the oscillating resistance, respectively, and *R*_dc_(*f*_0_) is the dc resistance measured for an input microwave source at a frequency *f*_0_. By considering trivial expressions from circuits theory, it is possible to compute the oscillating voltage at *f*_0_ and then ${\bar{Z}}_{{{{{{\rm{MTJ}}}}}}}\left(f\right)$ as:

6 $${\bar{Z}}_{{{{{{\rm{MTJ}}}}}}}\left({f}_{0}\right)=\left({R}_{{{{{{\rm{dc}}}}}}}\left({f}_{0}\right)-{R}_{{{{{{\rm{dc}}}}}}}(0)\right)+\frac{{I}_{{{{{{\rm{dc}}}}}}}{R}_{{{{{{\rm{M}}}}}}}{e}^{j\it\left({\varPhi }_{{{{{{\rm{R}}}}}}}-{\varPhi }_{{{{{{\rm{I}}}}}}}\right)}}{{I}_{{{{{{\rm{M}}}}}}}}$$

It can be observed that ${\bar{Z}}_{{{{{{\rm{MTJ}}}}}}}$ is given by two contributions: one is the change in the average resistance in the presence of the microwave input ${R}_{{{{{{\rm{dc}}}}}}}\left({f}_{0}\right)-{R}_{{{{{{\rm{dc}}}}}}}(0)$, and the other is proportional to the bias current and is related to the first harmonic of the resistance oscillations. Additional contributions, can be also added in the Eq. 6 in case of nonlinear resonance such as parametric excitations.

**Fig. 5:**
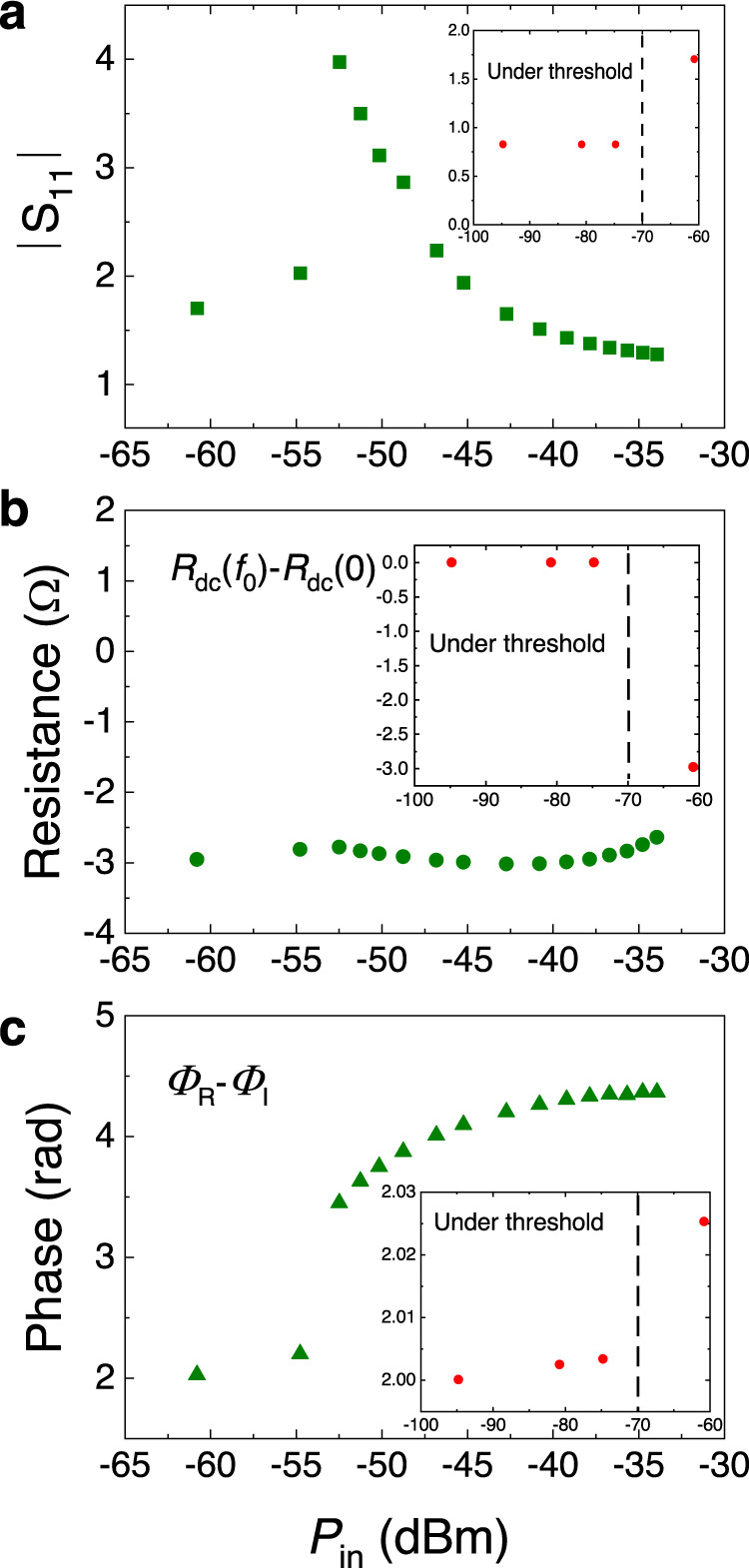
Micromagnetic simulations. **a** | S_11_ | calculated from the micromagnetic simulations by using Eq. (2) as a function of the input power in dBm. **b** and **c** Micromagnetic calculations of ${R}_{{{{{{\rm{dc}}}}}}}\left({f}_{0}\right)-{R}_{{{{{{\rm{dc}}}}}}}(0)$ and $\it{\varPhi }_{{{{{{\rm{R}}}}}}}-\it{\varPhi }_{{{{{{\rm{I}}}}}}}$ as a function of the input power *P*_in_ in dBm, the *R*_dc_ is the dc resistance, *Φ*_R_ − *Φ*_I_ is the phase shift. The insets of each panel show the calculations from – 60 to −100 dBm. The calculations have been performed for a current *J*_dc_ = −1.5 × 10^5 ^A/cm^2^. For this current density the self-oscillation excitation is sub-critical in deterministic simulations. In the inset we have also indicated the power at which the self-oscillation is not excited. The inset of each panel displays the data for the power range between −60 and −100 dBm.

Figure 5a shows the calculations of $\left|{\bar{S}}_{11}\left(f\right)\right|$ from the micromagnetic simulations as a function of the microwave current density amplitude *J*_M_ = *I*_M_/*S* (where *S* is the cross section of the MTJ) for the same magnetic field and voltage configuration of Fig. 2d. In particular, for the calculation of the ${\bar{S}}_{11}\left(f\right)$ we use micromagnetically computed values of ${R}_{{{{{{\rm{dc}}}}}}}\left({f}_{0}\right)-{R}_{{{{{{\rm{dc}}}}}}}(0)$ and $\left(\it{\varPhi }_{{{{{{\rm{R}}}}}}}-\it{\varPhi }_{{{{{{\rm{I}}}}}}}\right)$ as shown in Fig. 5b, c respectively. Note that the phase reported in Fig. 5c$\left(\it{\varPhi }_{{{{{{\rm{R}}}}}}}-\it{\varPhi }_{{{{{{\rm{I}}}}}}}\right)$ computed from micromagnetic simulations is the phase shift between the oscillating magnetoresistive signal and the input microwave current and it doesn’t coincide with the phase of *S*_11_ parameter (see Supplementary Fig. 8 for an example of the phase of *S*_11_ as a function of the input power).

In total, the micromagnetic simulations show a qualitative agreement with the experiments (Fig. 1d). In particular, those reproduce the non-monotonic trend of the |*S*_11_ | as a function of the microwave input power observed in the experiments. Micromagnetic simulations shown here are for a sub-critical bias current density *J*_dc_ = −1.5 × 10^5 ^A/cm^2^. Similar results with smaller value of |*S*_11_ | are also observed for larger current density where one needs larger microwave powers to observe the amplification. In addition, the multiple peaks excitation and the amplification mechanism are not related to the excitation of the large amplitude dynamics of the magnetization as shown in previous works^37,38^.

The appearance of the maximum is a consequence of the interplay of the positive first contribution in Eq. (6) and negative second one (recall that the amplification requires a negative impedance). At large microwave currents, the second contribution vanishes, as it is inversely proportional to the current. At low currents, it also vanishes because a low driving current induces low resistance oscillations (a small *R*_M_ in Eq. (6)) and, also, the phase difference between the resistance oscillations and microwave current changes; this indicates that STO is no longer in a perfect phase-locked state to the microwave drive due to the influence of the fluctuations (thermal in the experiment and numerical noise in the simulations). The micromagnetic simulations provide a larger value for the maximum |*S*_11_ | , which we attribute to the fact that the parasitic elements are not considered in our theoretical calculations.

To summarize, we demonstrate a spintronics amplifier based on MTJs and produced with CMOS-compatible materials that exhibit for some devices a gain larger than 2 at an input power down to the nW regime. This device configuration is designed in such a way that the dc voltage is applied to drive the free layer magnetization of the MTJ in a persistent dynamic state, i.e., to work as an STO. A bias field is applied along a direction that minimizes the STO nonlinearities, i.e., a nonlinear frequency shift that maximizes the STO output power. Compared to previous work, the mechanism driving a larger |*S*_11_ | , which measures the amplification in a two-terminal device, is purely electrical and take advantage of the nonlinear dynamics of spin-transfer-torque oscillators (STO) in presence of a low power microwave input where a partial injection locking mechanism is observed. This approach is not an incremental improvement of the amplifier performance, but it solves the problems arising from a heat-driven microwave amplification, which could cause undesirable side effects such as reduced fidelity.

We believe that these results will be stimulating for the community in the search of solutions which can have improved phase stability and then can be used for a wider range of applications. As we have shown, there are two main contributions to the ac output signal of our MTJ-based amplifier, which differently depends on the input power. Indeed, ac oscillations of resistance are proportional to magnetization oscillation amplitude *c*, while the variation of dc resistance is proportional to the square of *c* $\Delta {R}_{{{{{{\rm{dc}}}}}}}\sim {m}_{{{{{{\rm{\xi }}}}}}}^{2}$. Thus, contribution to the output voltage in Eq. (4) are proportional to ${m}_{{{{{{\rm{\xi }}}}}}}$ and ${m}_{{{{{{\rm{\xi }}}}}}}^{2}{I}_{{{{{{\rm{ac}}}}}}}$, where ${m}_{{{{{{\rm{\xi }}}}}}}={m}_{{{{{{\rm{\xi }}}}}}}\left({I}_{{{{{{\rm{ac}}}}}}}\right)\approx \sqrt{A+B{I}_{{{{{{\rm{ac}}}}}}}}$. By balancing between these contributions, it may be possible to design a large variety of amplification characteristics. In the two-terminal device studied here, it is hard to play with these contributions. Indeed, their relative impact is defined by the angle between the magnetizations of free layer and pinned layer (they are proportional to cos *φ* and sin *φ*, respectively), and this angle is hard to be tuned since it defines the spin-transfer-torque efficiency too. On the other hand, we believe, that much more flexibility could be achieved in a three-terminal device, in which input SOT-terminal is separated and, thus, STT-efficiency it is not affected by the angle between the free and pinned layer magnetizations. Additional advantages of a three-terminal device would be separation of input and output signals and possibility to match input and/or output impedances. Finally, the coupling of spintronic amplifiers with phase-locked loop circuits can potentially improve the phase stability by order of magnitudes.

**Fig. 6:**
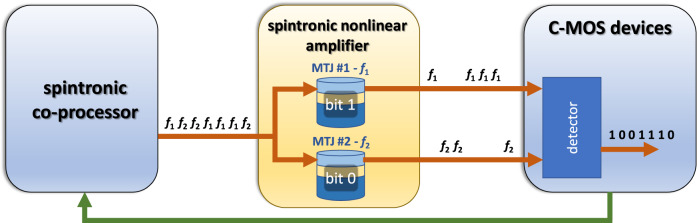
Concept of a main application of spintronic amplifiers in hybrid spintronic-CMOS systems. Ideally, the spintronic part of the circuit (spintronic co-processor) will work at power level below the CMOS part, and then the spintronic amplifiers will be used to transfer signal from the spintronic side to the CMOS side. The communication between them can be based on the implementation of an on-chip FSK modulation having bits coded in two frequencies (*f*_1_ and *f*_2_) and the two spintronic amplifiers centered in one of them respectively can amplify a signal at one of the two frequency which will be detected by the CMOS circuitry (spin diodes can be also used as proposed in ref. ^45^).

Our work provides an approach for the effective design of spintronic amplifiers for different applications. For example, in the Internet-of-Things nodes, those devices can be used as preamplifiers to improve the signal-to-noise ratio of weak microwave input signals. In neuromorphic computing, a chain of these oscillators can be used to implement long short-term memory or as a reservoir^39^, while for computing applications, it can be used as a building block for an Ising machine for the solution of combinatorial optimization problems^40–42^. In addition, spintronic microwave amplifiers should be necessary in hybrid CMOS-spintronic systems, where spintronic technology can be used as co-processor working at ultralow power level (nW or below). The spintronic nonlinear amplifier proposed here should amplify ultralow power signals of a sufficient amount to be reliably interfaced to CMOS technology as shown with a block diagram in Fig. 6^43^. It can be used for on-chip implementation of the Frequency Shift Keying (FSK) modulation realized for example with a tunable spintronic oscillator^44^. The bits are transmitted from the spintronic co-processor to the CMOS processing unit with a signal coding the information in different frequencies (*f*_1_ and *f*_2_) and then those signals are converted into a voltage by two MTJs, working as diodes, having a resonance frequency centered at those two frequency values^45^.

In conclusion, this device concept can potentially impact the realm of spintronics by opening different research directions. The first at the device level is the generalization of this two-terminal amplifier to a three-terminal MTJ combining spin-orbit torque (SOT) and STT. The bias voltage/current and input microwave power can be applied via the SOT terminal, while the output signal can be read via the magnetoresistive signal of the MTJ. These results can also impact the algorithmic level; in fact, an STO exhibiting amplification together with synchronization can be used as an efficient building block for neuromorphic computing and solving certain classes of combinatorial problems. Spintronic amplifiers can have a significant role in hybrid spintronic-CMOS systems where can be used to interface spintronic devices working at sub-nW power level to CMOS technology.

## Methods

### |*S*_11_ | measurements

The |*S*_11_ | values under *H*_ext,_ as shown in Figs. 1c and 2d were determined as follows: |*S*_11_ | was measured in the frequency range of 0.1 GHz < *f*  < 10.0 GHz, in the out-of-plane magnetic field range of −90 mT < *H*_ext_  <   −90 mT, in the azimuthal angle range of 0° < *θ* < 50° and in the polar angle range of 75° < *φ* < 110°.

### Time domain measurements

A 4-channel oscilloscope (Tektronix DPO73304D) 33 GHz electrical bandwidth is used in the new microwave setup (Supplementary Fig. 6), which has a sampling frequency of 50 giga-samples per second. The time-resolved voltage traces were recorded in Channel 1 and 2, respectively. When the signal generator turns off, the recorded voltage represents the STO microwave oscillation with free-running in the Channel 2. The RF signal provided by the signal generator was divided into two RF signals (RF_1_ and RF_2_) by the power divider. RF_1_ and RF_2_ have the same frequency, amplitude, and phase. RF_1_ signal is injected into Channel 1 of the Oscilloscope. RF_2_ signal, as the RF input signal, is applied into STO through a directional coupler and bias tee. Then, spin torque inducing microwave amplification voltage (RF output) was recorded in the Channel 2 of the Oscilloscope through the bias tee and directional coupler (Supplementary Fig. 6 and Supplementary Note 1).

### Micromagnetic simulations

We perform simulations with a finite difference integration scheme PETASPIN^46,47^. We consider an elliptical MTJ with the two axes with lengths of 130 nm and 60 nm and a free layer thickness *t*_FL_ = 1.65 nm. The discretization cell size is 4.0 × 4.0 × 1.65 nm^3^. The physical parameters saturation magnetization *M*_S_ = 950 kA/m, perpendicular anisotropy constant *k*_U_ = 0.52 MJ/m^3^, and magnetic damping *α* = 0.015 are identified experimentally, while the exchange constant *A* = 20 pJ/m is a typical value used for Co_20_Fe_60_B_20_. Qualitative similar results are also observed for *A* = 10 pJ/m. Details about the numerical implementation can be found in the Supplementary Note 2.

## Supplementary information

Supplementary Information

## Data Availability

The data that support the plots within this paper and other findings of this study are available from the corresponding author upon reasonable request.
