# Supplementary material for: Nonlinear amplification of microwave signals in spin-torque oscillators
Source: Nat Commun. 2023 Apr 17;14:2183. doi: 10.1038/s41467-023-37916-9 (PMC10110546; doi:10.1038/s41467-023-37916-9)
Supplement: Supplementary file 1 — Supplementary Information [file 41467_2023_37916_MOESM1_ESM.pdf]

Supplementary Materials for

Nonlinear amplification of microwave signals in spin-torque oscillators

Keqiang Zhu[1,8], Mario Carpentieri[2,8], Like Zhang[1,3,8], Bin Fang[1*], Jialin Cai[1], Roman Verba[4], Anna Giordano[5] ,Vito Puliafito[2], Baoshun Zhang[1], Giovanni Finocchio[6*], and Zhongming Zeng[1,7*]

[1]*Nanofabrication facility, Suzhou Institute of Nano-Tech and Nano-Bionics, Chinese Academy of Sciences, Suzhou, Jiangsu 215123, China*

[2]*Department of Electrical and Information Engineering, Politecnico di Bari, I-70125 Bari, Italy*

[3]*School of Electronics and Information Engineering, Wuxi University, Wuxi, Jiangsu 214105, China*

[4]*Institute of Magnetism, Kyiv 03142, Ukraine*

[5]*Department of Engineering, University of Messina, I-98166 Messina, Italy.*

[6]*Department of Mathematical and Computer Sciences, Physical Sciences and Earth Sciences, University of Messina, I-98166 Messina, Italy.*

[7]*Division of Nano-Devices and Technologies & Nanchang Key Laboratory of Advanced Packaging, Jiangxi Institute of Nanotechnology, Nanchang 330200, China*

[8]These authors contributed equally: Keqiang Zhu, Mario Carpentieri, and Like Zhang.

Supplementary Figure 1. Magnetoresistance measurement. Field scan of the magnetoresistive signal for an (a) in-plane magnetic field and (b) perpendicular magnetic field. The red and black arrows indicate the direction of the free layer and polarizer magnetization.

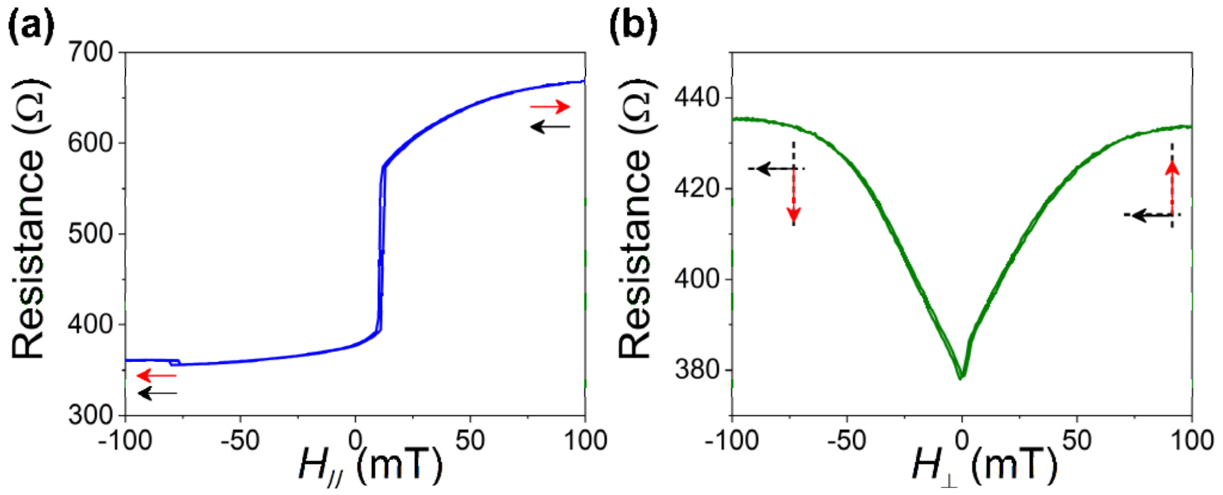

Supplementary Figure 2. **Microwave amplification with $|S_{11}|$ measurement for the device 1.** $|S_{11}|$ as a function of frequency (bias voltage V_{dc} = -400 mV) at different values of external field ranging from 30 to 90 mT for a device having the same nominal cross section of the device in the main text (device 1). Each panel is characterized by a different RF power, (a) -35 dBm, (b) -40 dBm, (c) – 45 dBm, and (d) -47 dBm.

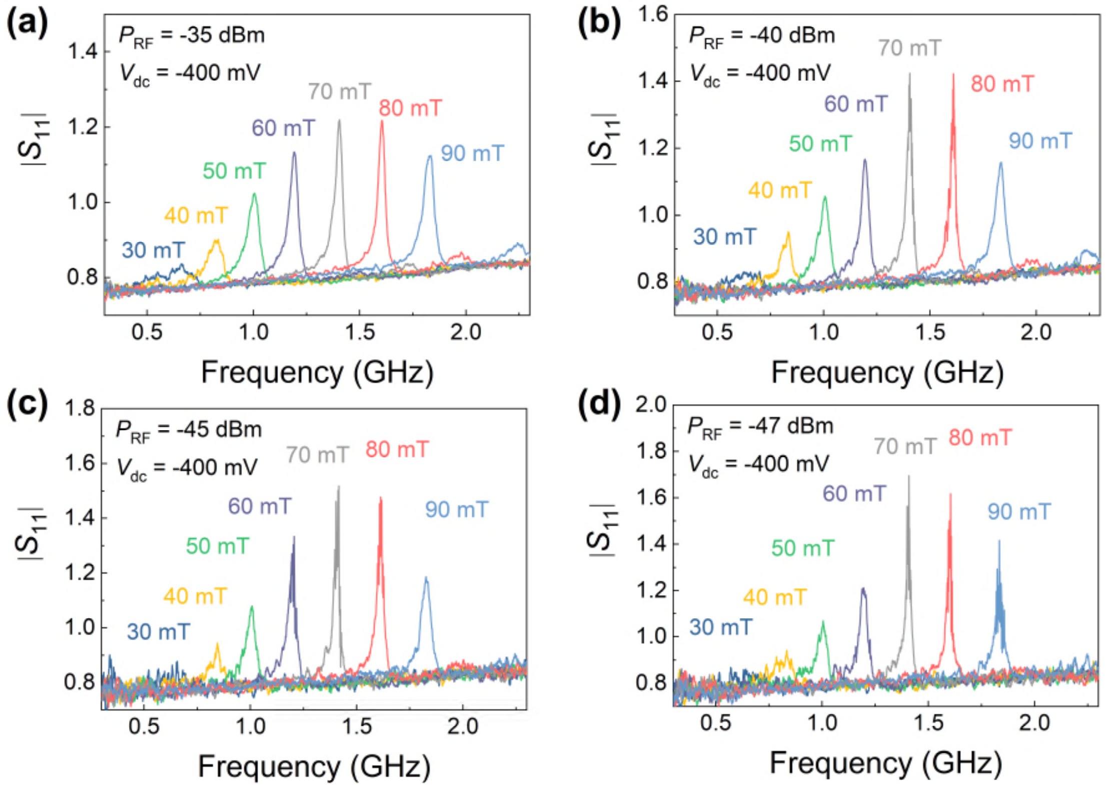

Supplementary Figure 3. Microwave amplification with $|S_{11}|$ measurement for the device 2. $|S_{11}|$ as a function of frequency (bias voltage V_{dc} = -400 mV) at different values of external field ranging from 30 to 90 mT for a device having same nominal cross section of the device in the main text (device 2). Each panel is characterized by a different RF power, (a) -35 dBm, (b) -40 dBm, (c) -45 dBm, and (d) -47 dBm.

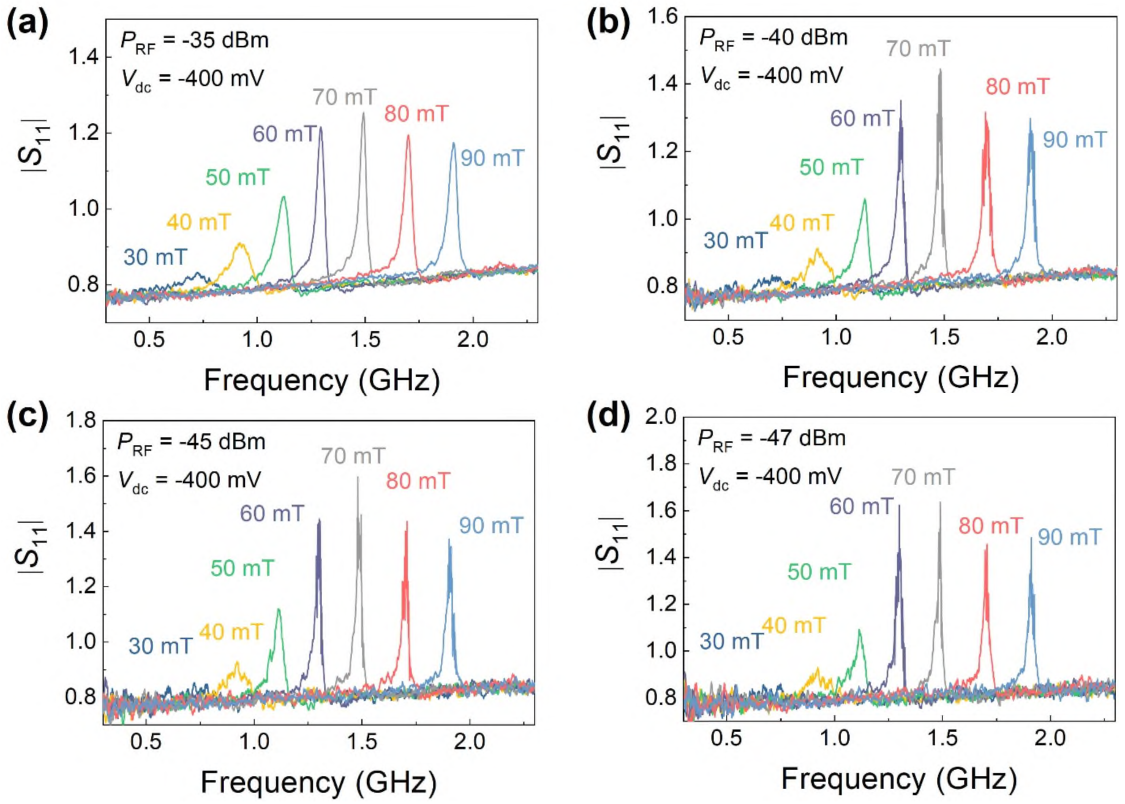

Supplementary Figure 4. |S_{11}| at different RF power. A comparison of |S_{11}| as a function of frequency (V_{dc} = -400 mV, H_{ext} = 600 Oe) for three values of external microwave, -60, -50 and -35 dBm. Those data are from the same device of the one shown in the main text.

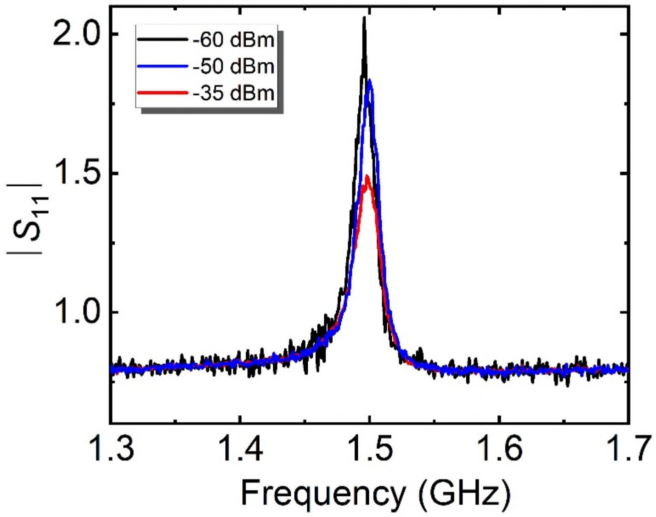

Supplementary Figure 5. Microwave emissions spectra at different input powers. The power ranges from -50 to -35dBm (f_{in}=1.498 GHz) power at H_{ext} = 60 mT and V_{dc} = -400 mV. **a** First and **b** second harmonics. An offset has been applied to separate the spectra. As can be observed at power approaching -37 dBm the second harmonics in the power spectra of the voltage becomes very large.

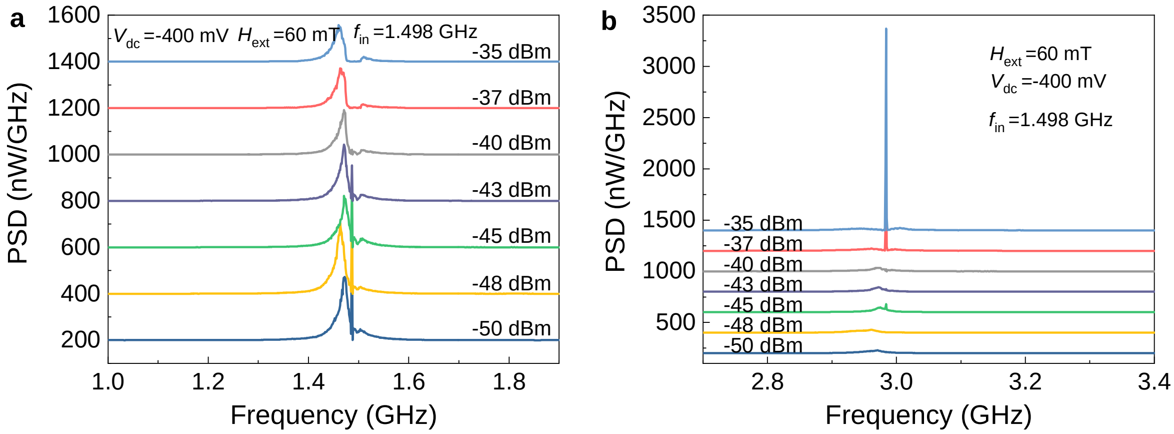

Supplementary Figure 6. The measurement circuit for the time-resolved voltage traces presented in this work. The RF signal provided by a signal generator is divided into RF_1 and RF_2. RF_1 and RF_2 have the same frequency, amplitude and phase. RF_1 is recorded by an oscilloscope in the channel 1. And RF_2 is the RF input applied into the STO through the directional coupler. The time-resolved voltage traces across the MTJ through the directional coupler is simultaneously measured in the channel 2 of the oscilloscope.

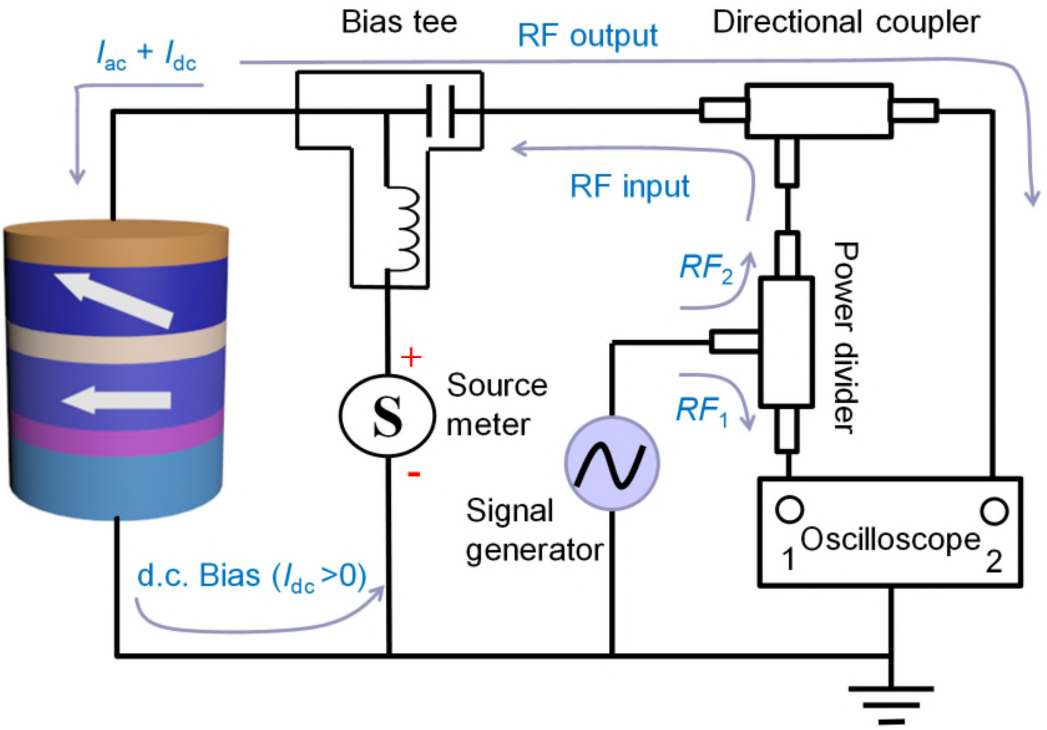

Supplementary Figure 7. The time domain measurements. (a) Time-resolved voltage traces for the STO in free running regime. Left panel shows 100 ns segments at I_{dc} = -0.46 mA for the magnetic field H_{ext} = 90 mT (θ = 30° and φ = 90°). Right panel is 40 ns window zoom of the previous trace. (b) Calculated time-drift of the oscillator phase from the data of the right panel in (a).

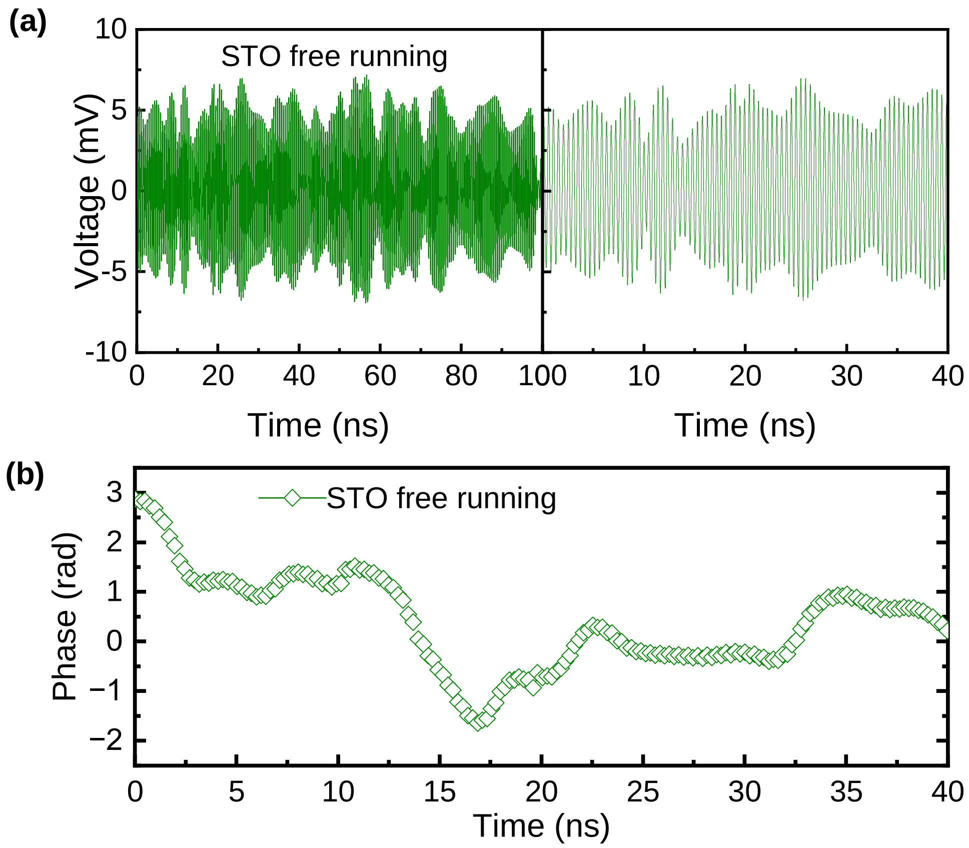

Supplementary Figure 8. An example of phase of the S_{11} scattering parameter as a function of the microwave input power.

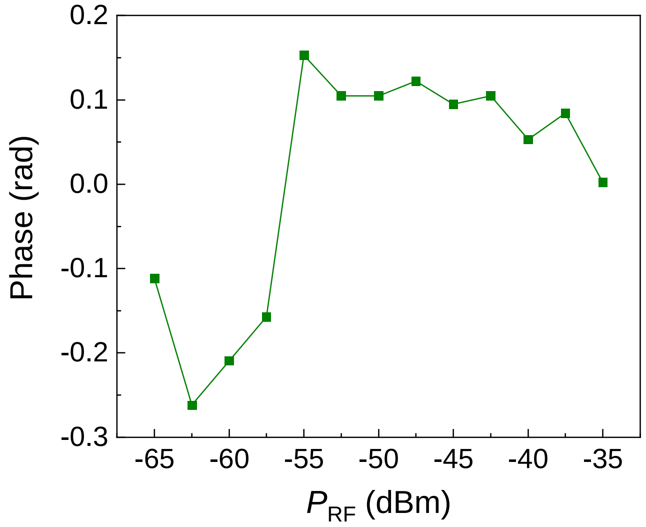

Supplementary Note 1. The time domain scheme and measurements

To characterize the phase coherence of the spintronic amplifier, we have used a different measurement setup which includes an oscilloscope to perform time-resolved voltage traces as shown in **Supplementary Figure 6.** The RF signal generated by a signal generator was divided into two RF signals (RF_1 and RF_2) by the power divider. RF_1 and RF_2 have the same frequency, amplitude and phase. RF_1 signal is injected into channel 1 of the oscilloscope. RF_2 signal, as RF input signal, is applied into nanoscale magnetic tunnel junction (MTJ) through a directional coupler and bias tee. When both the d.c. bias and RF_2 current were injected into the MTJ, spin torque inducing microwave amplification voltage (RF output) was measured and displayed in channel 2 through the bias tee and directional coupler.

First, we turned off the signal generator and applied the direct current I_{dc} = -0.46 mA (H_{ext} = 90 mT, θ = 30° and φ = 90°). **Supplementary Figure 7 (a)** displays a typical time-resolved voltage traces originated by the oscillating magnetoresistance in the STO. Note that STO is under free-running oscillation without interacting with the input signal.

To further evaluate the phase of the time-resolved trace, we calculate the phase of the oscillation at each zero crossing. (For the corresponding definition and more details see Ref. 1). The waveform may be written as

$$V(t) = [V_0 + \epsilon(t)]\sin[2\pi f_0 t + \emptyset(t)], \quad \text{(S1)}$$

where $\epsilon(t)$ is the deviation from the nominal amplitude V_0, f_0 is the nominal frequency, and $\emptyset(t)$ is the deviation from the nominal phase $2\pi f_0 t$. Note that $\emptyset(t)$ includes noise due to changes in precession amplitude combined with the coupling of amplitude and frequency in an STO. Zero crossing of $V(t)$ occur when $2\pi f_0 t + \emptyset(t) = n\pi$, where n is an even (odd) integer for crossings with a positive (negative) slope. The set of values $\{n_i, t_i\}$ for a waveform gives measurements of phase deviation at discrete times: $\emptyset(t_i) = 2\pi(n_i/2 - f_0 t_i)$. **Supplementary Figure 7 (b)** shows $\emptyset(t_i)$ for the span of 40 ns acquired with the time-resolved voltage traces of right panel of the **Figure 7 (a)**. The time domain phase illustrates some typical characteristics of the phase noise for the STO, which shows random walk character of the phase variations. Some phases show a relatively gradual change in about 1 rad between t = 3 ns and 11 ns and abrupt changes between t = 11 ns and 19 ns. While the phases keep nearly constant about -0.25 rad between t = 25 ns and 30 ns. As a results, phase noise with STO free-running dominates the spectral line broadening of STO, which influences drastically the coherence of the microwave signal broadening the linewidth[2].

Supplementary Note 2. Micromagnetic model.

The micromagnetic simulations are performed by using a state-of-the-art micromagnetic solver, PETASPIN, which numerically integrates the Landau-Lifshitz-Gilbert-Slonczewski (LLGS)[3,4]:

$$\frac{d\mathbf{m}}{d\tau} = -\left(\mathbf{m}\times\mathbf{h}_{\text{eff}}\right) + \alpha\left(\mathbf{m}\times\frac{d\mathbf{m}}{d\tau}\right) - \sigma\left[\mathbf{m}\times\left(\mathbf{m}\times\mathbf{m}_{\mathbf{p}}\right)\right] \tag{S2}$$

where $\mathbf{m} = \mathbf{M}/M_s$ is the normalized magnetization of the MTJ FL, $\tau = \gamma M_s t$ is the dimensionless time, γ is the gyromagnetic ratio, and M_S is the saturation magnetization. $\mathbf{h}_{\mathbf{eff}}$ is the normalized effective field, which includes the exchange, magnetostatic, anisotropic, external, and thermal fields. α is the Gilbert damping. $\sigma = \sigma_{\perp} / \left(1+\eta^2\cos\beta\right)$ [3,4] is the current-torque proportionality coefficient, where $\eta = 0.66$ is the spin polarization factor and $\beta = \arccos\left(\mathbf{m}\cdot\mathbf{m}_{\mathbf{p}}\right)$ is the angle between the FL magnetization and PL magnetization $\mathbf{m}_{\mathbf{p}}$, which is assumed to be completely fixed along the x-direction ($\mathbf{m}_{\mathbf{p}} = \hat{\mathbf{x}}$, with $\hat{\mathbf{x}}$ being the unit vector of the x-axis). $\sigma_{\perp} = \frac{2\eta g \mu_B j_P}{2\gamma e M_S^2 t_{FL}}$, where g is the Landé factor, μ_B is the Bohr magneton, *e* is the electron charge, and t_{FL} is the FL thickness. j_P is the current density flowing in the z-direction (see Inset of Fig. 1a), and $j_P = j_{\text{dc}} + j_{\text{ac}}\cos\left(2\pi f_{\text{ac}} t + \varphi\right)$, where j_{dc} is the bias current density while j_{ac} and f_{ac} are the amplitude and frequency of the current, respectively, and φ is the current phase.

We investigate an elliptical 130 nm × 60 nm MTJ with a free layer thickness t_{FL} = 1.65 nm as the experimental device and a discretization cell size of 4.0×4.0×1.65 nm^3. The physical parameters M_S = 950 kA/m, k_U = 0.52 MJ/m^3, and α = 0.015 are derived directly from the experimental measurement. The exchange constant *A* = 20 pJ/m is considered the same as that which we have used in our previous works for similar materials. However, simulations performed with *A* = 10 pJ/m give rise to qualitatively similar results. Micromagnetic simulations do not include thermal fluctuations.

References

1.Keller, M. W. et al. Time domain measurement of phase noise in a spin torque oscillator. *Appl. Phys. Lett.* **94**, 193105 (2009).

2.Kim, J. V. et al. Generation linewidth of an auto-oscillator with a nonlinear frequency shift: Spin-torque nano-oscillator. *Phys. Rev. Lett.*, **100**, 017207 (2008).

3.Lopez-Diaz, L. et al. Micromagnetic simulations using Graphics Processing Units. *J. Phys. D-Appl. Phys.* **45**, 323001 (2012).

4.Giordano, A. et al. Semi-implicit integration scheme for Landau-Lifshitz-Gilbert-Slonczewski equation. *J. Appl. Phys.* **111**, 07D112 (2012).
